# The pivotal role of the NLRC4 inflammasome in neuroinflammation after intracerebral hemorrhage in rats

**DOI:** 10.1038/s12276-021-00702-y

**Published:** 2021-11-30

**Authors:** Hui Gan, Li Zhang, Hui Chen, Han Xiao, Lu Wang, Xuan Zhai, Ning Jiang, Ping Liang, Shuyue Zheng, Jing Zhao

**Affiliations:** 1 Department of Pathophysiology, Chongqing Medical University, 400010 Chongqing, China; 2 Institute of Neuroscience, Chongqing Medical University, 400010 Chongqing, China; 3 Ministry of Education Key Laboratory of Child Development and Disorders, Children’s Hospital of Chongqing Medical University, 400010 Chongqing, China

**Keywords:** Molecular neuroscience, Acute inflammation

## Abstract

The NLRC4 inflammasome, a member of the nucleotide-binding and oligomerization domain-like receptor (NLR) family, amplifies inflammation by facilitating the processing of caspase-1, interleukin (IL)–1β, and IL-18. We explored whether NLRC4 knockdown alleviated inflammatory injury following intracerebral hemorrhage (ICH). Furthermore, we investigated whether NLRC4 inflammasome activation can be adjusted by the regulator of G protein signaling 2/leucine-rich repeat kinase-2 pathway. Fifty microliters of arterial blood was drawn and injected into the basal ganglion to simulate the ICH model. NLRC4 small interfering RNAs (siRNAs) were utilized to knockdown NLRC4. An LRRK2 inhibitor (GNE7915) was injected into the abdominal cavity. Short hairpin (sh) RNA lentiviruses and lentiviruses containing RGS2 were designed and applied to knockdown and promote RGS2 expression. Neurological functions, brain edema, Western blot, enzyme-linked immunosorbent, hematoxylin and eosin staining, Nissl staining, immunoprecipitation, immunofluorescence assay and Evans blue dye extravasation and autofluorescence assay were evaluated. It was shown that the NLRC4 inflammasome was activated following ICH injury. NLRC4 knockdown extenuated neuronal death, damage to the blood-brain barrier, brain edema and neurological deficiency 3 days after ICH. NLRC4 knockdown reduced myeloperoxidase (MPO) cells as well as tumor necrosis factor (TNF)-α, interleukin (IL)-6, IL-1β and IL-18 following ICH. GNE7915 reduced pNLRC4 and NLRC4 inflammasome activation. RGS2 suppressed the interaction of LRRK2 and NLRC4 and NLRC4 inflammasome activation by regulating pLRRK2. Our study demonstrated that the NLRC4 inflammasome may aggravate the inflammatory injury induced by ICH and that RGS2/LRRK2 may relieve inflammatory injury by restraining NLRC4 inflammasome activation.

## Brain hemorrhage: Reducing inflammation and brain damage

Treatments that target the proteins responsible for inflammation might reduce brain damage after a hemorrhage. Sudden bleeding into the brain, known as intracerebral hemorrhage (ICH), is associated with strokes and head injuries. Inflammation exacerbates ICH-induced injury after an incident, and is activated by protein complexes called inflammasomes. Jing Zhao and co-workers at Chongqing Medical University in China induced ICH symptoms in rats by extracting arterial blood and injecting it into the basal ganglia of the brain. When they artificially blocked expression of the inflammasome NLRC4, they saw reductions in neuron death, brain edema and damage to the blood-brain barrier. The team also identified a protein critical for activating NLRC4 and another protein that suppresses NLRC4. These findings could aid the development of treatments to block NLRC4 in the days after an ICH.

## Introduction

Intracerebral hemorrhage (ICH) is characterized by high mortality and high disability^1^, although it accounts for 10–15% of all stroke types^2^. A considerable amount of evidence has shown that intracerebral hemorrhage leads to a series of pathophysiological changes, including inflammation, edema, apoptosis, and necrosis^3^. Inflammation plays a key role in ICH-induced injury by releasing proinflammatory cytokines, especially interleukin (IL)-1β^4^. Increased expression of IL‐1β and IL‐18 is often observed upon brain injury^5^. The inflammasome, a part of the innate immune system, can cut pro-caspase-1 into cleaved caspase-1, which makes pro-interleukin-1β and pro-interleukin-18 mature and causes inflammatory responses^6^.

The nucleotide-binding and oligomerization domain-like receptor (NLR) family responds to innate immunity by forming inflammasomes^7^. NLRC4 (CARD12, IPAF) recruits pro-caspase‐1 directly through its CARDs without ASC (Pycard, PYD and CARD domain containing), although ASC contribute to the maturation of IL‐1β, cleaved caspase‐1 and IL‐18^8^. Moreover, phosphorylation at NLRC4-Ser^533^ is critical in the activation of the NLRC4 inflammasome^9^. The NLRC4 inflammasome is activated in bacterial inflammation^10^. However, neuroinflammation in ICH is sterile inflammation. The phosphorylation at NLRC4-Ser^533^ and the activation of the NLRC4 inflammasome after ICH remain to be elucidated.

Regulator of G protein signaling 2 (RGS2) consists of a single RGS domain with minimal flanking amino and carboxy-terminal regions^11^. The protective role of RGS2 in anxiety, panic disorder, and suicide has been reported^12^. Our previous study showed that RGS2 expression was upregulated and relieved inflammatory injury in a collagenase-induced intracerebral hemorrhage model^13^. However, the mechanism of the protective role of RGS2 in ICH remains unclear. Leucine-rich repeat kinase-2 (LRRK2) consists of GTPase domains, functional kinase domains, and multiple domains for protein–protein interactions^14^. LRRK2 is a well-known kinase closely related to Parkinson’s disease (PD)^15,16^. Intriguingly, Cui H and his colleagues have shown that LRRK2 kinase can activate the NLRC4 inflammasome in acute Salmonella typhimurium infection by promoting the phosphorylation of NLRC4 at Ser^533^ with interaction with NLRC4^17^. A report indicated that the level of phosphorylation of Ser935 can be used to assess the activity of LRRK2^18^. RGS2 modulates LRRK2 function by restricting the phosphorylation of LRRK2 in Parkinson’s disease^19^.

Therefore, our study aimed to investigate whether the NLRC4 inflammasome is activated by phosphorylation at NLRC4-Ser533 after ICH. We also explored whether LRRK2 aggravates the NLRC4 inflammasome and whether RGS2 regulates activation of the NLRC4 inflammasome after ICH via LRRK2.

## Materials and methods

### Rats

Sprague–Dawley rats (300–350 g, healthy, male and adult) were purchased from Chongqing Medical University with the license of the institutional animal care and use committee. All animal procedures were reviewed by the Institutional Animal Ethics Committee (IACUC) of Chongqing Medical University, Chongqing, China (NIH Publication No. 85–23, revised 1996). All animals used in this experiment were cared for in strict accordance with the Guide for the Care and Use of Laboratory Animals. All rats were housed under constant temperature (25–26 °C) with sufficient food and water. All efforts were made to relieve pain and unintentional death^20^.

### Experimental design

**Fig. 1:**
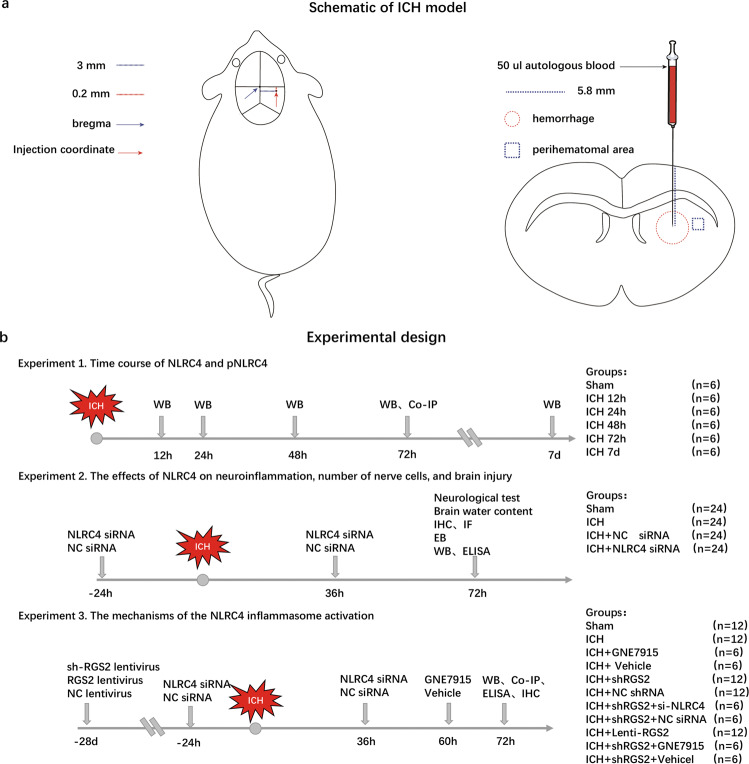
Schematics of ICH model and experimental design. **a** A schematic of the ICH model including indication of the stereotactic injection coordinates, hematoma volume, and the regions of the perihematomal area. **b** A schematic of the experimental design and animal groups.

A schematic of the experimental design, including the numbers of animals, groups and experimental methods, is shown in Fig. 1b.

### Experiment 1

To investigate the expression of NLRC4 and pNLRC4 and the interaction of NLRC4 with pro-caspase-1 after ICH, 36 rats were randomly divided into six groups: sham, 12, 24, 48, 72 h, and 7 d after ICH (*n* = 6). The samples of each group were used for Western blot (WB). In addition, the sham and ICH 72 h samples were shared for coimmunoprecipitation (Co-IP).

### Experiment 2

To explore the effects of NLRC4 on neuroinflammation, the number of nerve cells, and brain injury, 96 rats were randomly assigned to four groups: sham, ICH, ICH + NC siRNA and ICH + NLRC4 siRNA. Nine rats in each group were evaluated with a modified neurological severity score (mNSS). All rats were sacrificed at 72 h after ICH for brain water content (*n* = 6), immunohistochemistry (IHC)/immunofluorescence (IF) (*n* = 6), Evans Blue Dye Extravasation and Autofluorescence (EB) (*n* = 6), and Western blot (WB)/enzyme-linked immunosorbent assay (ELISA) (*n* = 6).

### Experiment 3

To explore the mechanisms of NLRC4 inflammasome activation, 66 rats were randomly divided into eleven groups: sham, ICH, ICH + GNE7915, ICH + vehicle, ICH + shRGS2, ICH + NC shRNA, ICH + shRGS2+si-NLRC4, ICH + shRGS2+NC siRNA, ICH + Lenti-RGS2, ICH + shRGS2 + GNE7915, and ICH + shRGS2 + vehicle. The samples of the sham, ICH, ICH + shRGS2, ICH + NC shRNA and ICH + Lenti-RGS2 groups were shared in Experiment 3. All rats were sacrificed at 72 h after ICH for Western blot (WB)/enzyme-linked immunosorbent assay (ELISA)/coimmunoprecipitation (Co-IP) (*n* = 6). An additional 30 rats were divided into five groups (sham, ICH, ICH + shRGS2, ICH + NC shRNA, ICH + Lenti-RGS2) for double immunofluorescence staining at 72 h after ICH (*n* = 6).

### ICH model

The rats first underwent intraperitoneal anesthesia with sodium pentobarbital. The surgical instruments were autoclaved, and Hamilton syringes and needles were sterilized with 84 disinfectants. Hamilton syringes and needles were rinsed in sterile water three times before use. The hair of the rats was removed, and the surface of the rats was wiped with 75% ethanol and allowed to dry. Then, 50 µL of autologous blood was taken from the femoral artery by a microinjection pump (Shenzhen Ruiwode Life Technology Co., Ltd., China) on the operation side with a microsyringe washed with heparin. Afterward, these rats were fixed on a stereotaxic apparatus (Shenzhen Ruiwode Life Technology Co., Ltd., China). The blood was automatically infused into the right basal ganglia within 5 min at 3.00 mm from the midline, 0.2 mm behind bregma, and 5.80 mm beneath the cortex. The needle was left for 10 min to prevent blood reflux before suturing the scalp. Throughout the experiment, the rats were placed in thermostatic blankets to keep the body temperature at approximately 37 °C. The sham group was only given a needle insertion. A schematic of the ICH model, including indication of the stereotactic injection coordinates, hematoma volume, and the regions of the perihematomal area, is shown in Fig. 1a.

### Brain water content

The brain-water content was assessed using the wet-dry method^21^. The brain was divided into the ipsilateral (hemorrhagic) side and contralateral side, weighed on an analytical microbalance and then dried at 100 °C for 48 h to determine the dry weight. The brain-water content (%) was measured accordingly (wet weight–dry weight)/wet weight × 100%.

### The modified neurological severity score

Motor, sensory (visual, tactile, and proprioceptive), balance and reflex tests were included in the modified Neurological Severity Score^22^. The neurological severity of the rats on day 3 after ICH was assessed by a score of 0 to 18, with 0 indicating a normal score and 18 indicating a maximum neurological deficit^23^.

### Hematoxylin and eosin (H&E) staining

After perfusion of normal saline from the heart, 4% paraformaldehyde was slowly infused into the whole body for internal fixation at 72 h after ICH. The brains were removed, fixed with 4% paraformaldehyde for 48 h, and dehydrated using 75, 80, 95 and 100% alcohol. Then, the brains were transparentized in dimethylbenzene, dipped in wax and cut into brain Section (5 μm thick) by a slicer. After dewaxing, debenzene, hematoxylin staining and eosin staining, the brain sections were dehydrated, cleared, and observed under a microscope.

### Nissl staining

The brain sections were made using the above method. After being debenzened and deparaffinized, brain sections underwent staining in a tar purple solution for 15 min before being washed in ddH_2_O. The brain sections underwent a color separation reaction and were then dehydrated in 70, 80, 95, and 100% ethanol. Finally, the sections were transparentized, mounted and observed under a microscope (×400 magnification). Neurons in the area around the hematoma were counted per microscopic field.

### siRNA transfections

NLRC4-siRNA was infused at a concentration of 2 μg/µL and retained for 10 min into the right lateral ventricle at 1 mm anterior–posteriorly, 2 mm mediolaterally, and 3.5 mm dorsoventrally^24,25^. siRNA-NLRC4 and scramble siRNA (si-NC or si-negative control) were transfected 24 h before and 36 h after ICH model establishment. Both were obtained from GenePharma (China): NLRC4-siRNA(sense: GCUGAGGCCCACGUAUAAATT; antisense: UUUAUACGUGGGCCUCAGCTT); Negative control siRNA(sense: UUCUCCGAACGUGUCACGUTT; antisense: ACGUGACACGUUCGGAGAATT).

### Inhibitor GNE7915 injection

DMSO (5%), 30% PEG 300, 5% Tween 80 and ddH_2_O were added to the inhibitor GNE-7915 at a concentration of 3.1 mg/mL GNE7915 and intraperitoneally injected (50 mg/kg) into the rats 60 h after ICH^26^.

### Lentivirus transfection

Rats were placed in a stereotactic frame (Shenzhen Ruiwode Life Technology Co., Ltd., China) after being anesthetized using 4% C_2_H_3_Cl_3_O_2_. The lentivirus vectors (Lenti-RGS2) for RGS2 overexpression and the short hairpin (sh) RNA (sh-RGS2) for RGS2 knockdown were produced (GenePharma Technology Corporation, PRC): sh-NC (sense: TTCTCCGAACGTGTCACGT); sh-RGS2 (sense: GCTCTGGGCAGAAGCATTTGA). Holes were made in the rat pericranium 1.9 mm behind the coronal suture and 0.9 mm from the sagittal suture. Then, a 10 μL microinjection pump was placed stereotactically 3.5 mm deeper under the cortex. Five microliters of lentivirus with 1 × 10^9^ genomic copies of Lenti-RGS2 and short hairpin (sh) RNA-RGS2 (sh-RGS2) were injected into the right lateral cerebral ventricle ipsilaterally at 0.5 μL/min^27^. Four weeks later, these rats underwent ICH surgery^28^.

### Co-Immunoprecipitation assay

To detect whether LRRK2 interacted with NLRC4, an LRRK2 antibody (1:80; Millipore Biologicals, USA) was incubated with magnetic beads to form a complex in solution. Then, the magnetic beads were separated, and the antibody was recycled. Next, the tissue sample was incubated with beads that would bind to the antibody to form an antibody/antigen complex, and the tissue sample was dissociated. To pull down the complex from the beads, loading buffer was diluted with PBS and added to the complex with the beads, which was subsequently abandoned. The LRRK2 proteins were separated by SDS–PAGE for Western blot analysis using anti-NLRC4 (1:80, Novus Biologicals, USA) to determine NLRC4. The same method was used to detect whether NLRC4 was combined with LRRK2 and whether NLRC4 interacted with pro-caspase-1.

### Immunofluorescence staining

Consecutive coronal sections of the brain (8 μm thick) were then blocked in 5% bovine serum albumin (BSA) at 37 °C for 1 h. They were incubated with rabbit anti-mouse MPO antibody (1:50, Abcam, USA), rabbit anti-mouse NLRC4 antibody (1:100; Novus Biologicals, USA), mouse anti-mouse LRRK2 antibody (1:100; Millipore Biologicals, USA), rabbit anti-mouse IBA-1 antibody (1:1000; Abcam, USA) and rabbit anti-GFAP antibody (1:200; Abconal, China) overnight at 4 °C. The samples were then incubated after three cycles of PBS washing with the corresponding fluorescence-conjugated secondary antibodies (1:100; Proteintech, USA) for 1 h at 37 °C. Three cycles of PBS washing were again performed. Microphotographs were analyzed with ImageJ software, and Pearson’s coefficient was used to evaluate the combination of NLRC4 and LRRK2.

### Western blotting

Proteins were loaded (50 μg), separated by 6, 8, and 15% SDS–PAGE and then transferred to polyvinylidene fluoride membranes (PVDF, Millipore, USA). The primary antibodies: rabbit anti-NLRC4 (1:800, Novus Biologicals, USA), mouse anti-pNLRC4 (1:400, ECM Biologicals, USA), mouse anti-RGS2 (1:500, Santa Cruz, USA), rabbit anti-Pro-caspase-1 (1:1000, Abconal, China), rabbit anti-Pro-IL-1β (1:1000, Abconal), rabbit anti-IL-18 (1:1000, Abconal, China), rabbit anti-cleaved caspase-1 (1:500, Cell Signaling, USA), rabbit anti-cleaved IL-1β (1:500, Affinity, USA), rabbit anti-cleaved IL-18 (1:200, R&D, USA), rabbit anti-TNF-α (1:500, Boster, China), rabbit anti-IL-6 (1:500, Boster, China), rabbit anti-LRRK2 (1:1000, Abcam, USA), rabbit anti-pLRRK2 s935 (1:500, Abcam, USA) and rabbit anti-β-actin (1:1000, Proteintech, USA). Secondary antibody (1:2000, Proteintech, USA)-linked horseradish peroxidase (HRP) was added to the bands for 2 h. ECL detection reagents (Thermo, USA) were used to visualize the bands. ImageJ software was used to determine the relative density of these proteins.

### Evans blue dye extravasation and autofluorescence

Evans blue dye (4%, 5 mL/kg, Sigma–Aldrich) was injected into the femoral vein under anesthesia. After 1 h, the rats were perfused with normal saline, and brain tissues were collected. Then, 50% trichloroacetic acid was put onto the brain tissues around the hematoma before the sample was homogenized and centrifuged at 12,000 × *g* for 30 min. The absorbance of the resulting supernatant was measured by a spectrophotometer at 620 nm. Meanwhile, the brains were cut into 8 µm slices by slicer (Leica, CM1860) and observed under a fluorescence microscope^29^.

### Enzyme-linked immunosorbent assay (ELISA)

Brain samples were harvested at day 3 after ICH, and the levels of IL-18 and IL-1β were examined with ELISA kits (Jiangsu Meibiao Biotechnology Co., Jiangsu, China).

### Statistical analysis

Data were described as the mean ± SD. GraphPad Prism software (version 7.0) was employed to conduct the statistical analyses. One-way ANOVA and Tukey’s multiple comparisons test were used to analyze parametric data. *P* < 0.05 indicated significant difference.

## Results

### The NLRC4 inflammasome is activated following ICH

**Fig. 2:**
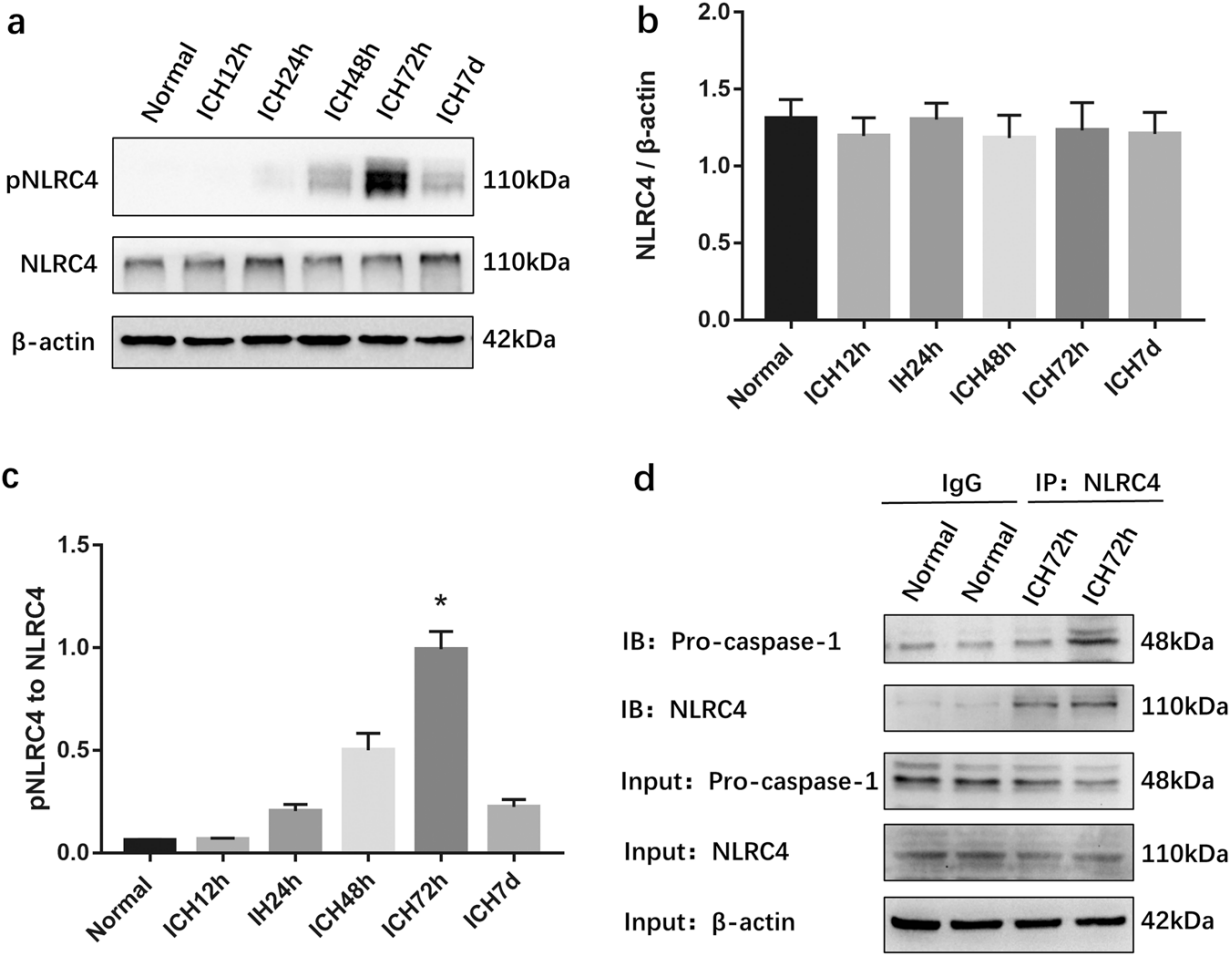
Expressions of NLRC4 and pNLRC4 after intracerebral hemorrhage, and the activation of the NLRC4 inflammasome. **a**–**c** NLRC4 and pNLRC4 expressions in the peri-hematoma area of sham and ICH rats were detected by Western blot at 12, 24, 48, 72 h, and 7 days after surgery, respectively (six rats for each group after ICH). *P < 0.05, compared with Sham group. **d** The results of Co-Immunoprecipitation showed the interaction of NLRC4 and pro-caspase-1 after ICH.

To investigate whether the NLRC4 inflammasome was activated after ICH, Western blotting was used to detect the levels of pNLRC4 and NLRC4, and coimmunoprecipitation was used to detect the interaction of NLRC4 with pro-caspase-1. The results indicated increased pNLRC4 levels after ICH, which reached a peak at approximately 72 h after intracerebral hemorrhage (Fig. 2a, c). There was no significant difference in NLRC4 levels between the six groups (Fig. 2a, b). The results of coimmunoprecipitation showed that NLRC4 interacted with and pro-caspase-1 in brain tissue at 72 h after ICH (Fig. 2d). These results revealed that ICH induced the phosphorylation of NLRC4 at Ser533 and activation of NLRC4 inflammasomes after ICH.

### NLRC4 promotes the accumulation of glial cells, neurological injury, brain edema, neuronal death, and damage to the blood-brain barrier following ICH

**Fig. 3:**
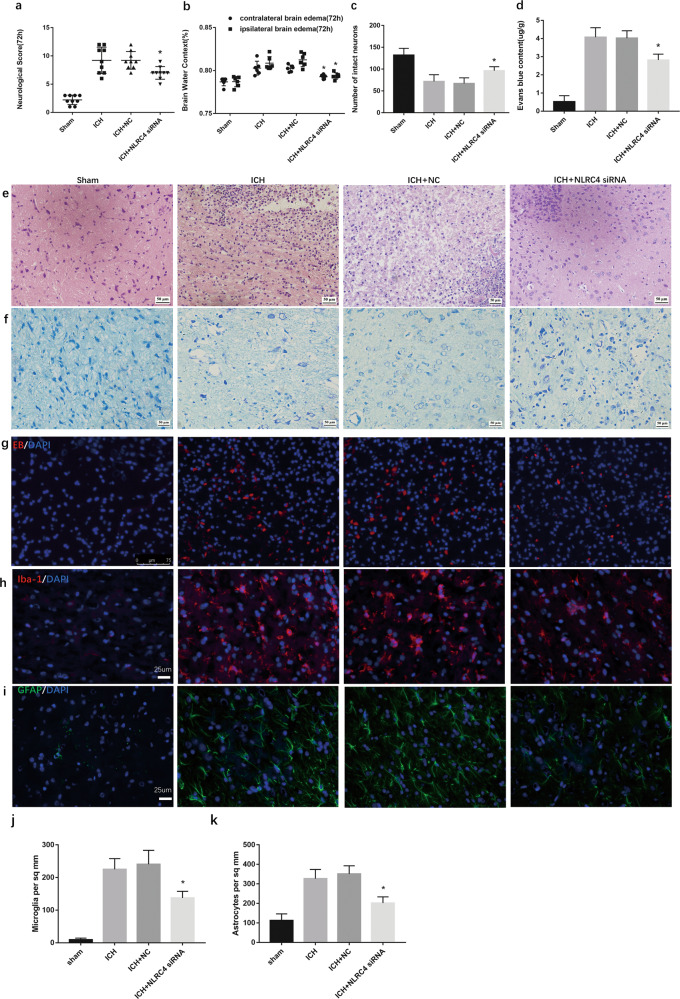
NLRC4 knockdown reduced accumulation of glial cells, neurological function damage and decreased brain edema after ICH. **a** Modified Neurological Severity Scores (mNSS) for neurological function, **b** Brain edema in ipsilateral brain and contralateral brain for brain water content, **e** HE staining for the morphology (×400), **f** Nissl staining for the morphology (×400) and **c** Nissl staining for the number of nissl bodies, **d** Evans blue Dye extravasation and **g** autofluorescence (×400) for the integrity of blood brain barrier, **h** IF of Iba-1 staining for microglia. **i** IF of GFAP staining for astrocytes. Quantification of microglial accumulation (**j**) and astrogliosis (**k**) in the Sham, ICH, ICH + NC, and ICH + NLRC4 siRNA groups at 72 h after ICH. The data represent the means ± SEM. (six rats for each group). *P < 0.05, compared with ICH.

To explore the role of NLRC4 in ICH, a specific and efficient siRNA targeting NLRC4 was designed. We subsequently used modified neurological severity scores (mNSSs) and brain water content to detect the role of NLRC4 in ICH-induced neurological injury. The results showed that, compared with the sham group, the ICH group at 72 h demonstrated severe neurological deficits and higher brain edema (Fig. 3a, b). Significant neurological improvement was seen in the NLRC4 siRNA+ICH group compared with the ICH group (Fig. 3a). After NLRC4 siRNA injection, brain edema in the ipsilateral brain and contralateral brain decreased significantly compared to that in the ICH group (Fig. 3b). Correspondingly, compared with the sham group, HE staining evidenced disorderly cytoplasmic loosening, karyopyknosis, and edema of the neurons in the ICH group. This alteration could be significantly reversed after treatment with NLRC4 siRNA (Fig. 3e). Nissl staining showed decreased Nissl bodies in the ICH group compared to the Sham samples. The number of Nissl bodies was significantly increased after treatment with NLRC4 siRNA (Fig. 3c, f). NLRC4 siRNA injection decreased EB leakage from blood vessels into the brain tissue at 72 h after ICH (Fig. 3d). The autofluorescence intensity (×400) of Evans blue declined with NLRC4 siRNA treatment (Fig. 3g). We tested the changes in microglial cells using the astrocyte marker GFAP and the microglial marker Iba-1 under this circumstance. We observed numerous accumulations of microglia and astrocytes after ICH (×400). There was a significant reduction in microglial accumulation in the ICH + NLRC4 siRNA group compared with the ICH group (Fig. 3h, j). Similarly, there was also a reduction in astrocytes in the ICH + NLRC4 siRNA group compared with the ICH group (Fig. 3i, k). These results indicated that NLRC4 exacerbates the accumulation of microglia and astrocytes after ICH. Altogether, these results showed that NLRC4 may aggravate ICH-induced brain injury in rats.

### NLRC4 facilitates neutrophil infiltration and the expression of TNF-α, IL-6, IL-1β, and IL-18 after intracerebral hemorrhage

**Fig. 4:**
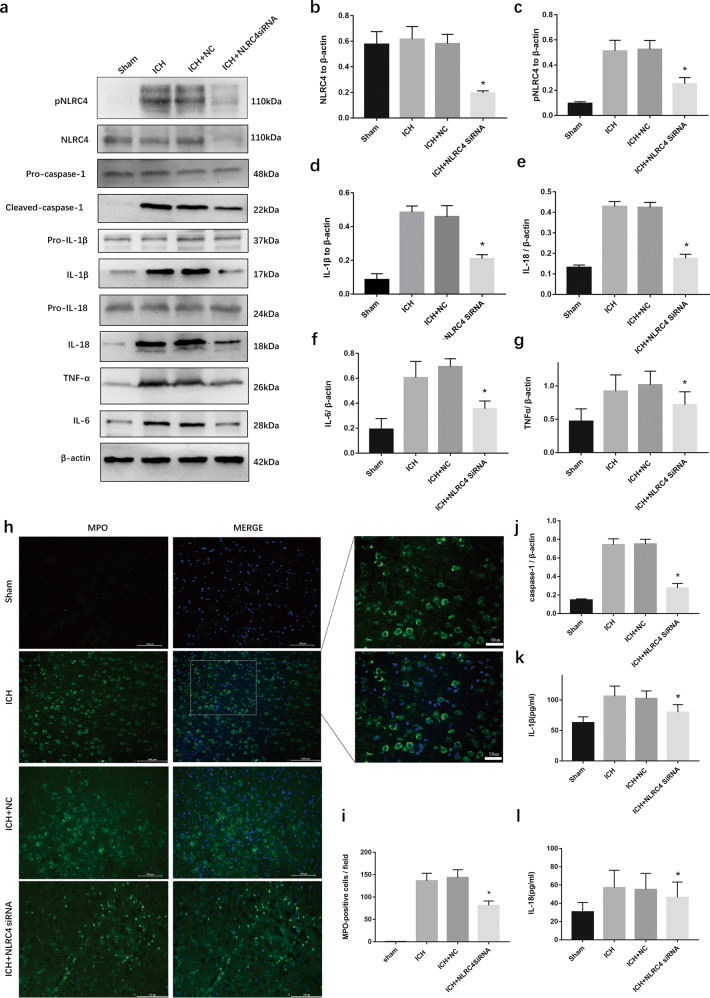
Inflammation response were reduced by NLRC4 siRNA after ICH. **a**–**g**, **j** Western blot assay to detect NLRC4, pNLRC4, Pro-IL-1β, IL-1β, Pro-caspase-1, caspase-1, Pro-IL-18, IL-18, TNF-α, and IL-6, (**k**, **l**) ELISA for IL–18 and IL–1β (**h**, **i**) Immunostaining for MPO-positive cells (×400 and ×200) at peri-hematoma area in sham, ICH, negative control siRNA, and NLRC4 siRNA groups at 72 h after ICH (six rats for each group). *P < 0.05, compared with ICH.

To explore the effect of NLRC4 on ICH-reduced inflammation, siRNAs were injected to knock down NLRC4. Western blot analysis revealed that the expression of NLRC4 and pNLRC4 was effectively reduced (Fig. 4a–c). Cleaved caspase-1, IL-1β and IL-18 levels were reduced by NLRC4 siRNA injection (Fig. 4a, d, e, j). Accordingly, ELISA showed that, compared with the ICH group, the levels of IL-18 and IL-1β were decreased (Fig. 4k, l) in the ICH + NLRC4 siRNA group. However, the expression of pro-caspase-1, pro-IL-1β and pro-IL-18 was not significantly different among the four groups. Additionally, TNF-α and IL-6 were also reduced by NLRC4 siRNA injection (Fig. 4a, f, g). We used immunostaining to detect neutrophil infiltration. Immunostaining (× 200 and × 400) results showed that NLRC4 siRNA treatment reduced MPO-positive cells around the hematoma compared to the ICH group (Fig. 4h, i). These data suggested that NLRC4 knockdown may mitigate neuroinflammation after ICH.

### RGS2 regulates the phosphorylation of NLRC4 and affects the NLRC4-dependent inflammasome activation

**Fig. 5:**
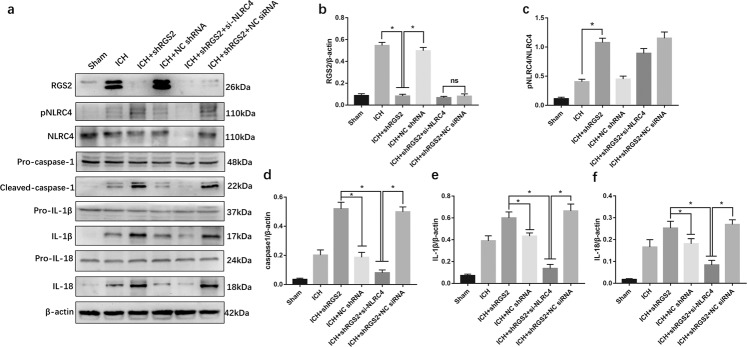
RGS2 affected NLRC4 inflammasome activation following ICH. **a**–**f** Western blot assay was used to detect the expression of RGS2, pNLRC4, NLRC4, Pro-caspase-1, caspase-1, Pro-IL-1β, IL-1β, Pro-IL-18, and IL-18, in perihematomal area in ICH, Sham, ICH + shRGS2, ICH + negative control shRNA (ICH + NC shRNA), ICH + shRGS2 + si-NLRC4 and ICH + shRGS2 + NC siRNA at 72 h after ICH (6 rats for each group).

To explore the effect of RGS2 on NLRC4 inflammasome activation, short hairpin (sh) RNA was used to knock down the expression level of RGS2, and siRNA was injected to knock down NLRC4. Since phosphorylation at NLRC4-Ser533 is critical in the activation of the NLRC4 inflammasome^9^, our results showed that shRNA-RGS2 increased the phosphorylation of NLRC4 at Ser533 but failed to reduce the expression of NLRC4 (Fig. 5a–c). In addition, shRNA-RGS2 increased the expression of IL-1β, caspase-1 and IL-18 (Fig. 5a, d–f). However, it seemed that shRNA-RGS2 failed to affect the expression of pro-caspase-1, pro-IL-1β, and pro-IL-18 (Fig. 5a). Moreover, siRNA-NLRC4 reversed the effect of shRNA-RGS2 on increasing the levels of IL-1β, caspase-1 and IL-18 (Fig. 5d–f). These results demonstrated that shRNA-RGS2 treatment significantly enhanced the phosphorylation of NLRC4 and the maturation of IL‐1β and IL-18 induced by NLRC4. Thus, RGS may regulate NLRC4 inflammasome activation.

### LRRK2 is critical for the activation of NLRC4 inflammasomes following ICH

**Fig. 6:**
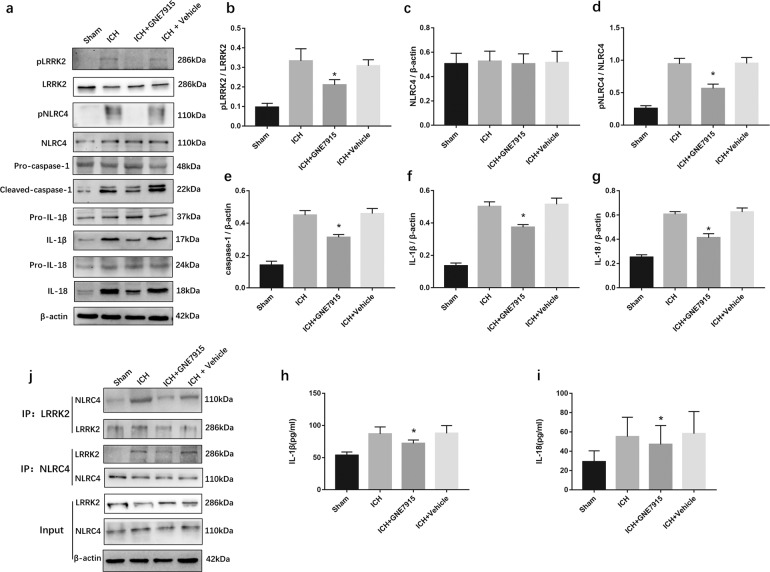
LRRK2 inhibitor extenuated NLRC4 inflammasome activation after ICH. **a**–**g** Western blot assay to detect LRRK2, pLRRK2, NLRC4, pNLRC4, pro- IL-1β, IL-1β, Pro- caspase-1, caspase-1, Pro-IL-18, and IL-18 (**h** and **i)** ELISA for IL-18 and IL-1β (**j**) Co-Immunoprecipitation assay for NLRC4 and LRRK2, in peri-hematoma area in sham, ICH, ICH + GNE7915, and ICH + Vehicle groups at 72 h after ICH (six rats for each group). *P < 0.05, compared with ICH.

LRRK2 is a certain and specific kinase that promotes the phosphorylation of NLRC4 and activates the NLRP4 inflammasome but not the NLRP3 inflammasome, accompanied by the formation of a complex with NLRC4 after S. Typhimurium infection^17^. Interestingly, it seems that the expression of LRRK2 did not change significantly after ICH. However, pLRRK2 at Ser935 was increased after ICH (Fig. 6a, b). This result indicated that LRRK2 may play a role dependent on its phosphorylation at Ser935 after ICH. We used GNE7915 (a specific inhibitor of pLRRK2) to verify whether pLRRK2 is needed for NLRC4 inflammasome activation following ICH.

GNE7915 effectively reduced the expression of LRRK2 Ser935 but failed to reduce the expression of NLRC4 (Fig. 6a–c). Of note, Western blot and ELISA results showed that GNE7915 decreased the phosphorylation of NLRC4 at Ser533 and attenuated the expression of caspase-1, IL-1β, and IL-18 following ICH (Fig. 6a, d–i). However, the expression of pro-caspase-1, pro-IL-1β, and pro-IL-18 was not significantly different among the four groups. These data indicated that LRRK2 promoted the phosphorylation of NLRC4 and the maturation of IL-1β and IL-18.

Since the NLRC4 and LRRK2 complex is required for NLRC4 inflammasome activation^17^. We next used coimmunoprecipitation to detect the interaction of NLRC4 and LRRK2. Our results revealed that LRRK2 interacted with NLRC4 in the ICH group. GNE7915 reduced the interaction of NLRC4 and LRRK2 (Fig. 6j). These results verified that LRRK2 regulated the phosphorylation of NLRC4 and the activation of the NLRC4 inflammasome following ICH.

### RGS2 may restrain NLRC4 inflammasome activation via LRRK2

**Fig. 7:**
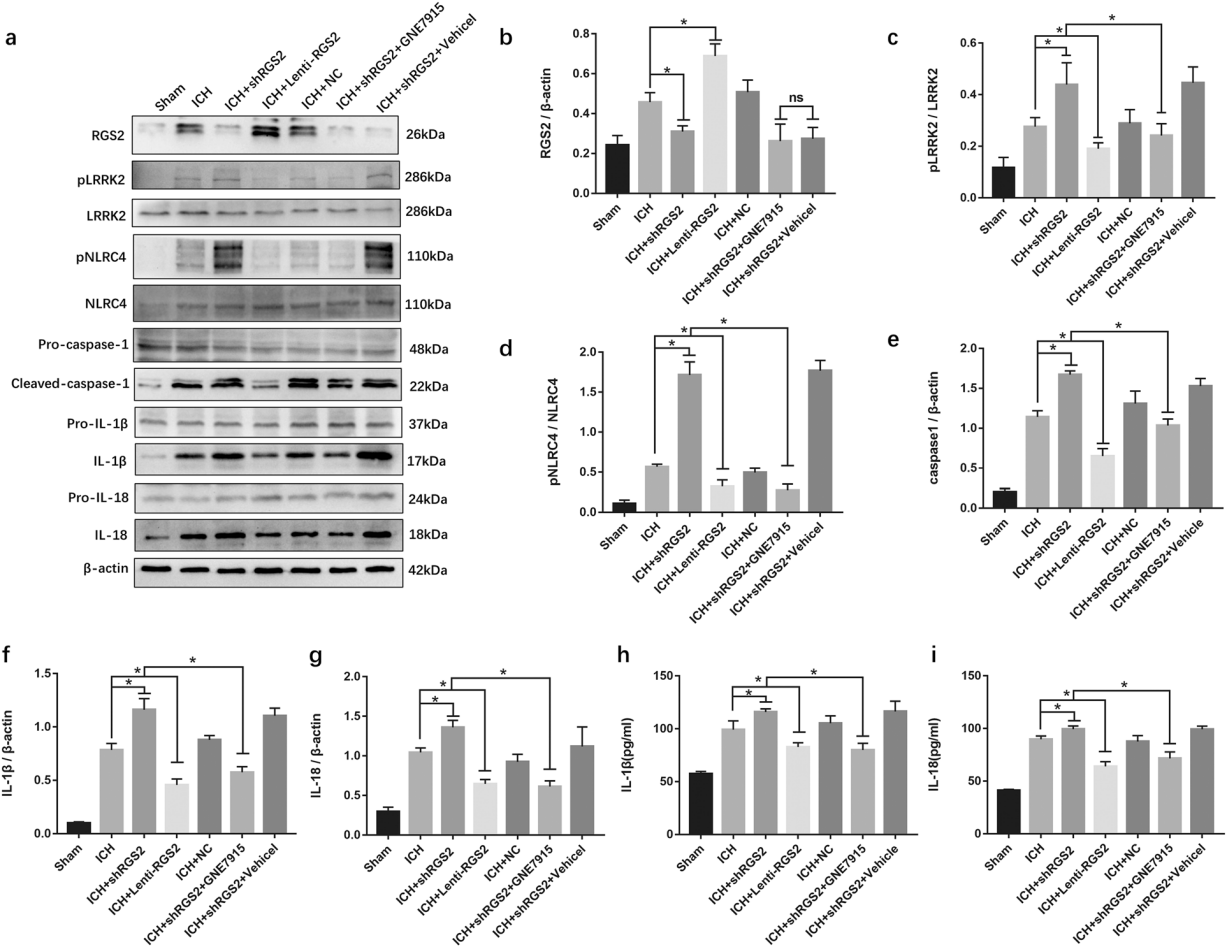
RGS2 affected NLRC4 inflammasome activation through regulating pLRRK2 following ICH. **a**–**g** Western blot assay was used to detect the expression of RGS2, LRRK2, pLRRK2, NLRC4, pNLRC4, pro- IL-1β, IL-1β, Pro-caspase-1, caspase-1, Pro-IL-18, and IL-18, **h**, **i** ELISA for IL-1β and IL-18 in perihematomal area in ICH, Sham, ICH + shRGS2, ICH + Lentil-RGS2, negative control lentiviral (ICH + NC), ICH + shRGS2 + GNE7915 and ICH + shRGS2 + Vehicle groups at 72 h after ICH (six rats for each group).

To determine how RGS2 affects NLRC4 inflammasome activation, short hairpin (sh) RNA was designed to knock down the expression level of RGS2, and lentivirus containing RGS2 was designed to overexpress RGS2. The Western blot results showed that Lenti-RGS2 reduced the phosphorylation of NLRC4 and the levels of IL-1β, caspase-1 and IL-18 (Fig. 7a, b, d–i). In contrast, shRNA-RGS2 enhanced the phosphorylation of NLRC4 and the expression of caspase-1, IL-1β, and IL-18 (Fig. 7a, b, d–i). The expression of pro-caspase-1, pro-IL-1β and pro-IL-18 was not significantly different in these seven groups. These results demonstrated that lenti-RGS2 treatment significantly decreased the phosphorylation of NLRC4 and the activation of the NLRC4 inflammasome, while treatment with shRNA-RGS2 had the opposite effect.

Next, we explored whether RGS2 affects NLRC4 inflammasome activation via LRRK2; notably, RGS2 failed to regulate the expression of LRRK2 (Fig. 7a). We found that Lenti-RGS2 reduced the phosphorylation of LRRK2; conversely, shRNA-RGS2 enhanced the phosphorylation of LRRK2 (Fig. 7a–c). Then, we used GNE7915 to reduce pLRRK2. Our results found that GNE7915 reversed the effect of shRNA-RGS2 on increasing the phosphorylation of NLRC4 and the expression of IL-1β, caspase-1 and IL-18 (Fig. 7a, b, d–i). The results demonstrated that RGS2 may regulate the phosphorylation of LRRK2 and that RGS2 may restrain NLRC4 inflammasome activation via pLRRK2.

### RGS2 regulates the interaction of NLRC4 and LRRK2

**Fig. 8:**
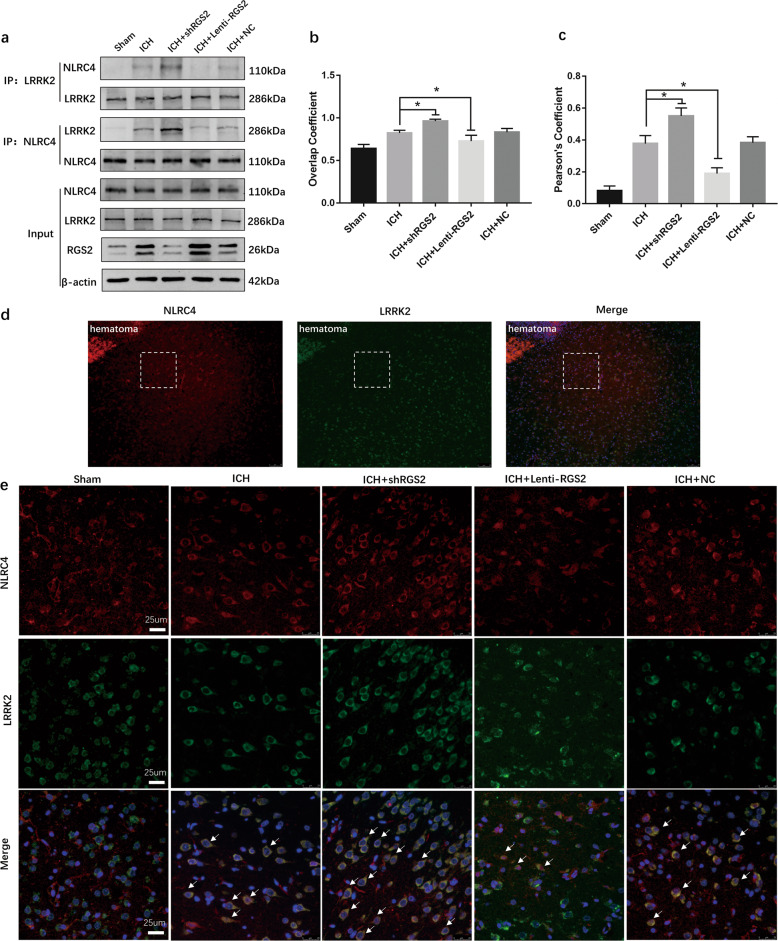
RGS2 affected the interaction of NLRC4 and LRRK2 following ICH. **a** Co-Immunoprecipitation assay for NLRC4 and LRRK2, **d** The location of the image around the perihematomal area **e** Representative images of Immunofluorescence (IF) colocalization for NLRC4 and LRRK2. **b**, **c** Pearson’s coefficient and overlap coefficient for NLRC4 and LRRK2 in peri-hematoma area in ICH, Sham, ICH + shRGS2, ICH + Lentil-RGS2 and negative control lentiviral (ICH + NC) groups at 72 h after ICH (six rats for each group). *P < 0.05, compared with ICH.

Since the NLRC4 and LRRK2 complex is required for NLRC4 inflammasome activation, our aforementioned results showed that LRRK2 interacted with NLRC4 after ICH (Fig. 6j). Next, we detected the effect of RGS2 on the interaction of NLRC4 and LRRK2. Coimmunoprecipitation results revealed that Lenti-RGS2 prevented the interaction of LRRK2 and NLRC4; conversely, shRNA-RGS2 promoted the interaction of LRRK2 and NLRC4 (Fig. 8a). The immunofluorescence colocalization results (Fig. 8d, e) were consistent with the coimmunoprecipitation results. The Pearson’s coefficient and overlap coefficient were lower in the ICH + Lenti-RGS2 group than in the ICH group (Fig. 8b, c). Conversely, the Pearson’s coefficient and overlap coefficient were higher in the ICH + shRGS2 group than in the ICH group (Fig. 8b, c). These results suggested that RGS2 may regulate the interaction of LRRK2 and NLRC4 during intracerebral hemorrhage.

## Discussion

The pathogenesis of ICH-induced brain injury includes primary brain injury and secondary brain injury (SBI). Increasing evidence has shown that SBI is a key factor in the deterioration of neurological function after ICH^30^. SBI consists of inflammation, oxidation, autophagy, and apoptosis^31^, leading to destruction of the blood-brain barrier and massive neuronal cell death^32^. Inflammation induced by the innate immune system plays an important role in SBI after ICH^33,34^. The NLR family, intracellular innate immune sensors, is responsible for processing pro-IL-1β and pro-IL-18 into a maturation state to promote inflammation^35,36^. The role of the NLR family in neuroinflammation is well known, especially NLRP1 and NLRP3. However, there are currently few studies on the role of NLRC4 in neuroinflammation. Our data revealed that NLRC4 could be activated by recognizing a damage-associated molecular pattern (DAMP) sensor after ICH. In this study, we found that NLRC4 reached the peak of phosphorylation at Ser533 on the 3rd day and that the NLRC4 inflammasome was activated after ICH, which corresponded to the peak of the proinflammatory cytokine IL-1β at 2–3 days after ICH^37^. A study on NLRC4^−/−^ mice after ischemic stroke demonstrated that the NLRC4 inflammasome contributes to acute brain injury^38^. Moreover, the NLRC4 inflammasome is reported to promote alcohol-induced liver injury^39^ and breast cancer progression^40^. Similar to these studies, our results showed that NLRC4 knockdown reduced brain damage. Furthermore, NLRC4 knockdown also reduced the expression of TNF-a and IL-6. Meanwhile, neutrophil infiltration, neuronal cell death and blood-brain barrier injury were alleviated in NLRC4 knockdown rats. These results might be related to NLRC4 playing a role in inflammation independent of inflammasome activation^41^. Overall, our results indicated that NLRC4 may result in the aggravation of intracerebral hemorrhage-related inflammation.

It is relatively clear that increased release of Il-1β and IL-18 from microglia has been identified as an important cytokine in ICH pathology^42,43^. Inhibitors and antagonists of IL-1β and IL-18 could extenuate brain edema, neuroinflammation, and neurodegeneration in experimental rats^44,45^. Furthermore, our results indicated that NLRC4 promoted the release of the cytokines IL-1β and IL-18, accumulation of microglia, neuronal death, Evans blue extravasation blood–brain barrier permeability, and brain water content for brain edema (Figs. 3 and 4). Taken together, we speculated that NLRC4 might promote all these outcomes via NLRC4 inflammasome-derived IL-1β and IL-18 processing. As mentioned before, both IL-1β and IL-18 play a crucial role in brain edema, neuroinflammation, and neurodegeneration^44,45^. Our experiment showed that IL-1β and IL-18 were decreased, and both may be involved in ICH injury. However, a previous study indicated that both IL-1β and IL-18 are required for NLRC4-derived inflammation, but IL-1β is more effective than IL-18 in treating bone erosion in Nlrc4-H443P-Tg Mice^46^. As far as the current evidence, we could not conclude whether IL-1β or IL-18 is more effective in NLRC4-induced inflammation after ICH.

We concluded that NLRC4 inflammasomes are involved in intracerebral hemorrhage-induced inflammation. Nevertheless, the molecular mechanisms of NLRC4 inflammasome activation in ICH are poorly known. GNE7915, a highly selective and BBB-penetrable LRRK2 inhibito^47^, was used to decrease the phosphorylation of LRRK2. We found that GNE7915 reduced pLRRK2 at Ser935 and the phosphorylation of NLRC4. Accordingly, IL-1β, caspase-1, and IL-18 levels were also decreased by GNE7915, as expected. Many studies are consistent with our results. George T and his colleagues reported that LRRK2 may result in neuronal apoptosis after cerebral ischemia by modulating the phosphorylation of Tau^48^. Similarly, LRRK2 contributes to traumatic brain injury (TBI)-induced neuronal apoptosis, BBB permeability, brain edema and neurological impairment^49^. The phosphorylation of LRRK2 may be related to NLRC4 inflammasome activation after ICH.

LRRK2, due to its complex structure, is also a Roco protein. It is an atypical G-protein that is a member of the Ras/GTPase superfamily called the Ras of complex proteins (Roc)^50^. It has been shown that Rab GTPase activity is regulated by GEF, GAP and GDI proteins^51^. RGS2 has been reported as a physiological GTPase activation protein (GAP) of LRRK2^19^. RGS2 has been regarded as a controller of GPCR and linked G protein signaling. RGS terminates the signal by speeding the intrinsic activity of GTPase in the G protein, which returns the G protein to the receptor in its GDP-bound form (inactive)^52^. RGS2 was considered as a key regulator of AHR^53^. This resulted from the specific interaction between RGS2 and G proteins, which was dependent on the linked GPCR’s selective recognition^54^. RGS2 was also reported to play a protective role in airway inflammation by reducing the number of granulocytes (neutrophils and eosinophils) and the release of inflammatory cytokines and chemokines^55^. Similarly, we discovered that RGS2 exerted a protective role in the neuroinflammation induced by the NLRC4 inflammasome following ICH by regulating LRRK2, which may not be related to the phosphorylation of LRRK2. These findings indicated that blocking NLRC4 may be a novel potential therapeutic target after ICH.

**Fig. 9:**
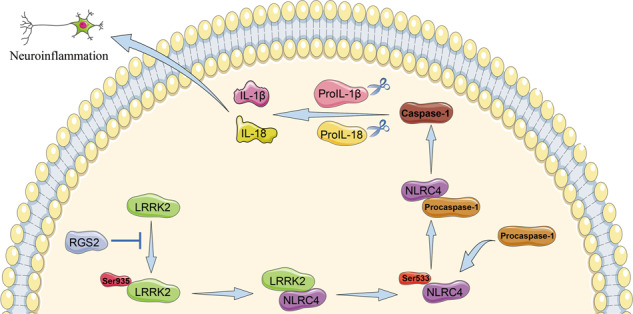
The role of the NLRC4 inflammasome in neuroinflammation after ICH. A pattern diagram showed that the NLRC4 inflammasome plays a role in ICH-induced neuroinflammation and that RGS2 regulates NLRC4 inflammasome activation by LRRK2 after ICH.

In summary, our study indicated that the NLRC4 inflammasome may play a role in ICH-induced inflammatory activation by increasing neutrophil infiltration and the expression of TNF-α, IL-6, IL-1β, and IL-18. Furthermore, our research provided novel insights into the NLRC4 inflammasome after intracerebral hemorrhage and suggested the role of RGS2 in NLRC4 inflammasome activation by LRRK2 after ICH (Fig. 9). It also identified RGS2 and LRRK2 as targets for the NLRC4 inflammasome in ICH.
